# Validation of EuroSCORE II in patients undergoing coronary artery bypass grafting (CABG) surgery at the National Heart Institute, Kuala Lumpur: a retrospective review.

**DOI:** 10.12688/f1000research.14760.2

**Published:** 2019-09-10

**Authors:** Ahmad Farouk Musa, Xian Pei Cheong, Jeswant Dillon, Rusli Bin Nordin

**Affiliations:** 1 Jeffrey Cheah School of Medicine and Health Sciences, Monash University Malaysia, Bandar Sunway, Selangor, Malaysia; 2 Department of Cardiothoracic Surgery, National Heart Institute , Kuala Lumpur, Malaysia

**Keywords:** EuroSCORE II, predictor, coronary artery bypass graft, post-CABG mortality, National Heart Institute

## Abstract

**Background**: The European System for Cardiac Operative Risk (EuroSCORE) II was developed in 2011 to replace the aging EUROScore for predicting in-house mortality after cardiac surgery. Our aim was to validate EuroSCORE II in Malaysian patients undergoing coronary artery bypass graft (CABG) surgery at our Institute.

**Methods**: A retrospective single-center study was performed. A database was created to include EuroSCORE II values and actual mortality of 1718 patients undergoing CABG surgery in Malaysia from 1st January to 31st December 2016. The goodness-of-fit of EuroSCORE II was determined by the Hosmer-Lemeshow goodness-of-fit test and discriminatory power with the areas under the receiver operating characteristics (ROC) curve (AUC).

**Results:** Observed mortality rate was 4.66% (80 out of 1718 patients). The median EuroSCORE II value was 2.06% (Inter Quartile Range: 1.94%) (1st quartile: 1.45%, 3rd quartile: 3.39%). The AUC for EuroSCORE II was 0.7 (95% CI 0.640 – 0.759) indicating good discriminatory power. The Hosmer-Lemeshow goodness-of-fit test did not show significant difference between expected and observed mortality in accordance to the EuroSCORE II model (Chi-square = 13.758, p = 0.089) suggesting good calibration of the model in this population. Cross-tabulation analysis showed that there is slight overestimation of EuroSCORE II in low-risk groups (0-10%) and slight underestimation in high-risk groups (>20%). Multivariate logistic regression analysis showed that gender, age, total hospital stay, serum creatinine and critical pre-operative state are significant predictors of mortality post-CABG surgery.

**Conclusion**: This study indicated that the EuroSCORE II is a good predictor of post-operative mortality in the context of Malaysian patients undergoing CABG surgery. Our study also showed that certain independent variables might possess higher weightage in predicting mortality among this patient group. Therefore, it is suggested that EuroSCORE II can be safely used for risk assessment while ideally, clinical consideration should be applied on an individual basis.

## Introduction

Coronary Artery Bypass Grafting (CABG) surgery, being a major surgery, is not without significant risks, up to and including death. In the United States, operative death rate and in-hospital mortality rate post CABG between 1997 and 2001 ranged from 1% to 5% for all patients ^1, 2^. In Malaysia, statistics from the National Heart Institute (IJN) had shown that the mortality rate for patients undergoing CABG surgery in Malaysia was around 2.7% ^3^. Notwithstanding, it is important to take note that the associated risk is very much dependent on multiple interacting factors including patients’ comorbidities and occurrence of any complications due to the operation itself ^4, 5^.

The need for a simple tool to predict post-surgical mortality led to the development of the European System for Cardiac Operative Risk Evaluation (EuroSCORE), also known as the European System for Cardiac Operation Risk Evaluation in 1999. This is a risk evaluation tool to calculate and predict operative mortality in patients undergoing cardiac surgery. It was developed using risk factors collected from almost 20,000 patients from more than 100 hospitals in Europe ^6^.

Since the publication of EuroSCORE, it had been widely employed and validated in various populations of cardiac surgical patients. However, it was found that the additive score for EuroSCORE tended to underestimate the risk of mortality, possibly when there were co-existing risk factors in high-risk patients. These concerns led to the development of the more complicated logistic EuroSCORE I. This version did produce a better estimate of risk in high risk patients. However, its main drawback is the overestimation of risk despite improvements in cardiac surgical outcomes observed ^7^.

In order to overcome this issue, the EuroSCORE team has come up with a revised version, which is known as the EuroSCORE II during the 2011 EACTS meeting in Lisbon. EuroSCORE II was developed by collecting and analysing prospective risk and outcome data on 22,381 patients undergoing major cardiac surgery in 154 hospitals in 43 countries over a 12-week period (May–July 2010). The new EuroSCORE II has updated the definition of renal function and unstable angina. Also, it further subdivided the classification of pulmonary hypertension, urgency and weight of operation. Most importantly, the new model has also changed the definition of outcome measurement, from 30-day mortality rate to in-hospital mortality. The main reason was the loss of follow-up data after discharge in certain centres, thereby giving rise to poor quality data in the original EuroSCORE ^8^.

Throughout the years, multiple validation studies have been conducted around the world including Europe, America and Asia to examine the validity of EuroSCORE II in predicting post-operative mortality and it had shown different results regarding the discriminatory power and calibration of this scoring system in different populations.

Furthermore, the EuroSCORE II has yet to be validated in Malaysia, a country with high incidence of cardiovascular diseases. Therefore, this study will serve as the first in Malaysia to examine the validity of EuroSCORE II in predicting operative mortality among patients undergoing CABG surgery.

## Methods

### Study design

A single-centre retrospective review was conducted at the National Heart Institute (IJN), the largest heart center in Malaysia. Almost all of the information needed was retrieved from the IJN electronic in-house database. Out-of-hospital data including death and late complications was obtained via telephone enquiry.

### Ethical statement

Ethical approval was obtained from both the IJN Research Ethics Committee (IJNREC/238/2018) and Monash University Human Research Ethics Committee (MUHREC/12981). The study was also registered with the National Medical Research Register (NMRR-17-2749-39322).

### Study sample

Within the period from 1 ^st^ January 2016 to 31 ^st^ December 2016, 1718 consecutive patients undergoing CABG surgery at the IJN were included in this study.

Inclusion Criteria: All patients undergoing CABG including two or three procedures.

Exclusion Criteria: Reinterventions for any cause in the same admission as the primary operation.

### Measurements

EuroSCORE II included ten patient-related factors, five cardiac-related factors, and three operation-related factors. Patient related factors include age (year), gender (male/female), renal impairment (creatinine clearance), extracardiac arteriopathy, poor mobility, previous cardiac surgery, chronic lung disease, active endocarditis, critical preoperative state and diabetes on insulin. Cardiac related factors include the New York Heart Association (NYHA) stages, Canadian Cardiovascular Society (CCS) class 4 angina, Left Ventricular (LV) function (ejection fraction > 50%, 31-50%, 21-30%, <20%), recent myocardial infarction (MI) (within 90 days) and pulmonary hypertension (31-55 mm Hg / >55 mm Hg). Operation related factors include urgency (elective, urgent, emergency, salvage), weight of the intervention (isolated CABG, isolated single non-CABG, 2-procedures, 3-procedures) and surgery on thoracic aorta. Details regarding EuroSCORE II calculation are available from the EuroSCORE site. The outcome variable, which is in-hospital mortality, was retrieved from the in-hospital database. In other words, it simply means death occurring at any time after surgery during the current admission. Additionally, important clinical information including presence of comorbidities (hypertension and hypercholesterolemia), total hospital stay, total ICU stay and follow-up status were also collected. A database is then created to collect the relevant data and stored in spreadsheets.

### Statistical analysis

Data was evaluated using the Microsoft Excel 2016 database (Microsoft Inc.) and analyzed using the Statistical Package for Social Sciences (SPSS) version 23.0. Continuous variables were presented as mean and standard deviation. Categorical variables were presented as frequencies and compared between groups using the chi-square test. A multiple logistic regression analysis was undertaken to determine significant predictors of in-hospital mortality. Predictive ability of the estimation model was assessed through discriminatory power and calibration. Receiver operating characteristics (ROC) curve analysis was performed to estimate the discriminant ability of this risk scoring model in predicting immediate post-operative mortality. It was considered good if the area under the curve (AUC) was >0.70. Calibration was evaluated using the Hosmer-Lemeshow goodness-of-fit test.

## Results

### Patients’ backgrounds

The demographics and pre-operative characteristics of patients are shown in Table 1. In terms of social demographics, mean age was 60 ± 8.89 years old, women made up 15.9% of the total sample, and Malay constituted the largest ethnic group (53.8%), which corresponds to the race distribution in Malaysia. Majority of the patients had comorbidities such as hypertension (83.3%) and hypercholesterolemia (77.4%). Preoperatively, the majority of patients were in NYHA class I (41.2%) and II (49.9%). Majority (46.6%) had good left ventricular function. Intraoperatively, majority of patients underwent isolated CABG (86.6%) without previous history of cardiac surgery (98.7%). In terms of weight of intervention, there are 160 patients who underwent a combination of two procedures, which included CABG + AVR, CABG + MVR as well as CABG + Aortic Root Replacement. For 33 patients who underwent three procedures, it included CABG+MVR+Aortic Root Replacement; CABG+MVR+AVR; as well as CABG + ASD Closure + Devega’s Tricuspid Annuloplasty.

**Table 1.** Patients’ demographics, preoperative and
intraoperative variables (n=1718).

Variables	
Age, year; mean ± SD (Range)	60 ± 8.89 (28 - 88)
Gender Male Female	1444 (84.1%) 274 (15.9%)
Race Malay, n (%) Chinese, n (%) Indian, n (%) Others, n (%)	924 (53.8%) 373 (21.7%) 333 (19.4%) 88 (5.1%)
Hypertension, n (%)	1431 (83.3%)
Diabetes Mellitus, n (%)	968 (56.3%)
Diabetes on insulin, n (%)	349 (20.3%)
Hypercholesterolemia, n (%)	1329 (77.4%)
NYHA NYHA I, n (%) NYHA II, n (%) NYHA III, n (%) NYHA IV, n (%)	707 (41.2%) 857 (49.9%) 117 (6.8%) 8 (0.5%)
Left ventricular function (EF: Ejection Fraction) EF > 50%, n (%) EF 31 – 50%, n (%) EF 21 – 30%, n (%) EF < 21%, n (%)	801 (46.6%) 716 (41.7%) 132 (7.7%) 21 (1.2%)
Types of CABG On-pump Off-pump	1687 (98.2%) 30 (1.7%)
Extracardiac arteriopathy, n (%)	70 (4.1%)
Poor mobility, n (%)	41 (2.4%)
Previous cardiac surgery, n (%)	22 (1.3%)
Chronic lung disease, n (%)	101 (5.9%)
Active endocarditis, n (%)	7 (0.4%)
Critical pre-operative state, n (%)	81 (4.7%)
CCS class IV angina, n (%)	26 (1.5%)
Recent MI, n (%)	552 (32.1%)
Pulmonary hypertension PA Systolic: < 31mm Hg, n (%) PA Systolic: 31 – 55mm Hg, n (%) PA Systolic: > 55mm Hg, n (%)	1647 (95.9%) 60 (3.5%) 11 (0.6%)
Urgency Elective, n (%) Urgent, n (%) Emergency, n (%) Salvage, n (%)	1518 (88.4%) 168 (9.8%) 32 (1.9%) 0 (0%)
Weight of intervention Isolated CABG, n (%) Isolated, single non-CABG, n (%) Two procedures, n (%) Three procedures, n (%)	1488 (86.6%) 4 (0.2%) 160 (9.3%) 33 (1.9%)
Surgery on thoracic aorta, n (%)	12 (0.7%)
EuroSCORE II; median (range)	2.06 (0.5 – 45.3)

n: Number of patients, SD: Standard deviation, CCS: Canadian Cardiovascular Society, MI: Myocardial infarction, NYHA: New York Heart Association, EF: Ejection fraction, CABG: Coronary artery bypass grafting, PA: Pulmonary artery, EuroSCORE: European system for cardiac operative risk evaluation

### Observed and predicted in-hospital deaths

The actual in-hospital mortality rate was 4.7% (80 out of 1718 patients). In comparison, predicted mortality rate by the median EuroSCORE II value was 2.06 (1 ^st^ quartile: 1.452, 3 ^rd^ quartile: 3.389). In other words, the predicted in-hospital mortality rate was slightly lower compared to the observed mortality rate. The correct classification was seen for 1638 out of 1718 patients, giving rise to a success rate of 95.3%. Actual mortality rate, by quartiles of EuroSCORE II, was 1.6% in the first quartile, 3.0% in the second quartile, 4.7% in the third quartile and 9.4% in the fourth quartile as shown in Table 2.

**Table 2.** Actual in-hospital mortality according to EuroSCORE II quartiles.

Outcome	Quartiles of EuroSCORE II			
	[0 – 1.45] %	[1.46 – 2.05] %	[2.06 – 3.39] %	[>3.39] %
Alive	422 (98.4%)	421 (97%)	408 (95.3%)	387 (90.6%)
Died	7 (1.6%)	13 (3%)	20 (4.7%)	40 (9.4%)
Total	429	434	428	427

EuroSCORE: European system for cardiac operative risk evaluation

### Discriminatory power

**Figure 1.**
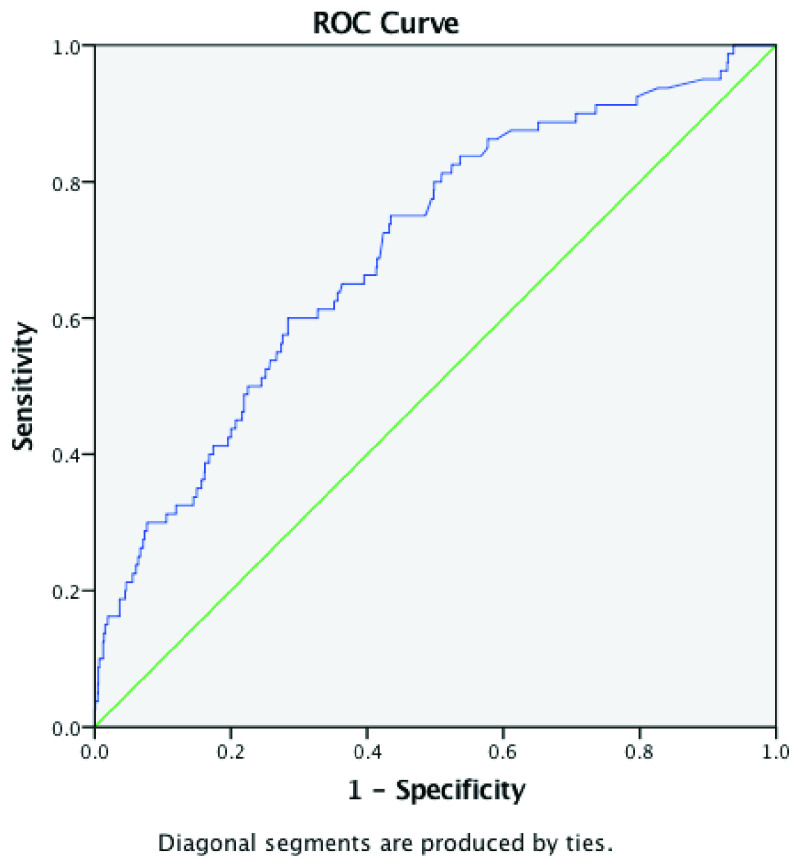
Receiver operating characteristic (ROC) curve for EuroSCORE II of 1718 CABG patients (Area Under the ROC curve = AUC= 70.0%) EuroSCORE: European system for cardiac operative risk evaluation.

As illustrated in Figure 1, the area under the receiver operating characteristic curve (AUC) was 0.7 (95% CI 0.640 – 0.759, p < 0.001), suggesting that the EuroSCORE II has fair and acceptable discriminatory power to discriminate between incidences of patients who died and those who were alive.

### Calibration (predictive power)

**Figure 2.**
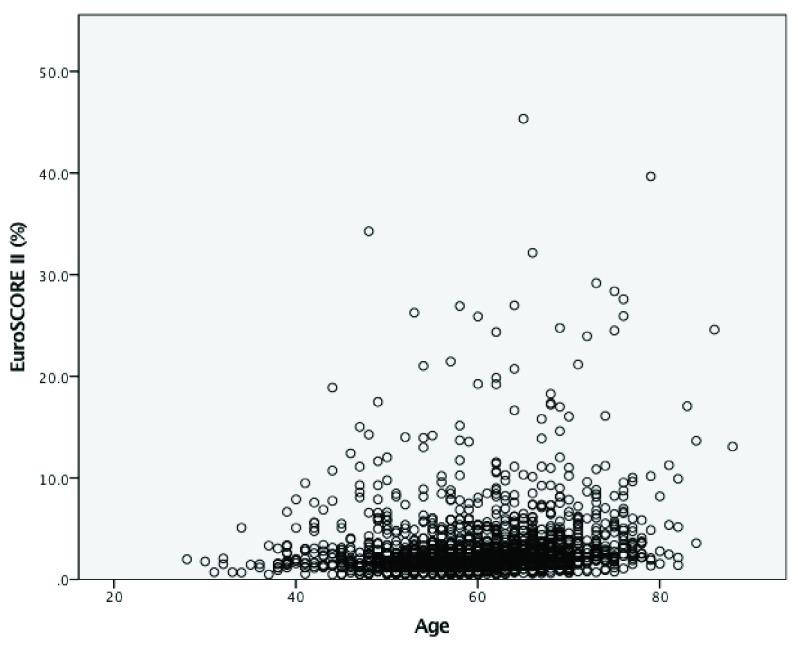
Scatter plot of EuroSCORE II of the 1781 CABG patients, by age (in years).

The Hosmer-Lemeshow (HL) goodness-of-fit test did not show significant difference between expected and observed mortality in accordance to the EuroSCORE II model (Chi-square: 13.748, *p* = 0.089), indicating reasonable calibration of this model in predicting in-hospital mortality among patients who underwent CABG surgery. Cross-tabulation analysis of predicted risk by EuroSCORE II showed that there was slight overestimation in low risk group (0 – 10%) and slight underestimation in high risk group (>20%) as shown in Table 3. Among the 8 patients who died in the 11-20% subgroup, 4 of them had isolated CABG (50%), while 2 of them underwent two procedures (25%) and the other 2, three procedures (25%) respectively. Figure 2 shows the relationship between age and EuroSCORE II in patients post-CABG in the IJN, Malaysia.

**Table 3.** Cross-tabulation analysis of EuroSCORE II based on estimated risk classes (0–10%, 11–20%, >20%).

Predicted Risk	Patients who died		Patients who were alive		Total patients
	Observed	Expected	Observed	Expected	
0 – 10%	67	76.9	1585	1575.1	1652
11 – 20%	5	2.1	40	42.9	45
>20%	8	1	13	20	21

EuroSCORE: European system for cardiac operative risk evaluation

### Subgroup analysis

Analysis was subsequently performed based on weightage of procedures. Among 1488 patients who underwent isolated CABG, we observed an actual in-hospital mortality rate of 3.9% and a median EuroSCORE II (predicted mortality) of 1.918, which showed an underestimation of risk. Hosmer and Lemeshow Goodness-of-Fit test showed a significant p value of 0.032, indicating a significant difference between expected and actual mortality among this group of patients. Discriminatory power as shown by the ROC curve analysis showed an area of 67.8%.

Among 160 patients who underwent two procedures, we observed an actual in-hospital mortality rate of 10% and a median EuroSCORE II (predicted mortality) of 3.712, which showed an underestimation of risk. However, Hosmer and Lemeshow Goodness-of-Fit test showed a non-significant p-value of 0.591, indicating no significant difference between expected and actual mortality among this group of patients. Discriminatory power as shown by the ROC curve analysis showed an area of 63.4%.

Lastly, among 33 patients who underwent three procedures, we observed an actual in-hospital mortality rate of 18.2% and a median EuroSCORE II (predicted mortality) of 6.2, which showed an underestimation of risk. Similar to two procedures, Hosmer and Lemeshow Goodness-of-Fit test showed a non-significant p-value of 0.575, indicating no significant difference between expected and actual mortality among this group of patients. Discriminatory power as shown by the ROC curve analysis showed an area of 67.9%.

Analysis on discriminatory power were repeated on other subgroups, including gender, race (Malay, Chinese, Indian and others), age (below 60 years old, 60 years old and above) and presence of comorbidities (hypertension, diabetes mellitus and hypercholesterolemia). Subgroup analysis showed that AUC for the EuroSCORE II was 0.695 in male (95% CI 0.620 – 0.770, *p* < 0.001), 0.642 in female (95% CI 0.534 – 0.751, *p* = 0.017), 0.696 in Malay (95% CI 0.624 – 0.767, *p* < 0.001), 0.801 in Chinese (95% CI 0.696 – 0.906, *p* < 0.001), 0.642 in Indians (95% CI 0.470 – 0.813, *p* = 0.083), 0.596 in other ethnicities (95% CI 0.153 – 1.000, *p* = 0.573), 0.700 in those aged below 60 years old (95% CI 0.571 – 0.830, *p* = 0.006), 0.673 in those age 60 years old and above (95% CI 0.603 – 0.743, *p* < 0.001), 0.691 in hypertensive patients (95% CI 0.628 – 0.754, *p* < 0.001), 0.745 in patients without hypertension (95% CI 0.585 – 0.905, *p* = 0.04), 0.672 in diabetic patients (95% CI 0.602 – 0.741, *p* < 0.001), 0.728 in patients without diabetes (95% CI 0.614 – 0.841, *p* < 0.001), 0.683 in patients with hypercholesterolemia (95% CI 0.615 – 0.750, *p* < 0.001) and 0.768 in patients without hypercholesterolemia (95% CI 0.650 – 0.886, *p* < 0.001).

### Independent variables analysis

Multivariate binary logistic regression analysis was undertaken to develop a prediction model of variables in EuroSCORE II and outcome (in-hospital mortality). The forward conditional method was selected to be used for analysis. The last step showed that being female, aged more than or equal to 65 years old, serum creatinine more than 120 micromole/litre and longer ICU stay are significant and independent predictors of in-hospital mortality in patients undergoing CABG surgery as shown in Table 4. The model which consists of the four risk factors explained between 24.6% (Cox and Snell R-square) and 47.8% (Nagelkerke R-square) of variance in predicting in-hospital mortality among patients undergoing CABG surgery in Malaysia. Moreover, it correctly classified 99% of the cases. These independent variables also made a unique statistically significant contribution to the model (χ2 (8) = 92.403, p < 0.001).

**Table 4.** Multiple logistic regression analysis showing association between age, gender, serum creatinine, and length of intensive care unit (ICU) stay (independent variables) and in-hospital mortality (dependent variable).

Variables	*p* value	Adjusted Odds Ratio	95% CI	
			Lower	Upper
Age ≥ 65 years	0.015	3.381	1.273	8.984
Female	0.037	3.279	1.076	10.000
Serum Creatinine ≥ 120 µmol/L	0.012	3.429	1.306	9.000
ICU stay (day)	<0.001	4.170	3.107	5.598

Dataset 1. Demography and EUROScore II variables (Excel) http://dx.doi.org/10.5256/f1000research.14760.d202215
Copyright: © 2019 Musa AF et al. 2019 ${data-license-text}

Dataset 2. Demography and EUROScore II variables (SPSS) http://dx.doi.org/10.5256/f1000research.14760.d202216
Copyright: © 2019 Musa AF et al. 2019 ${data-license-text}

Dataset 3. SPSS Output http://dx.doi.org/10.5256/f1000research.14760.d202217
Copyright: © 2019 Musa AF et al. 2019 ${data-license-text}

## Discussion

Accurate prediction of risk is always essential and plays an important role in guiding doctors to make clinical decision as to whether surgery is an appropriate intervention, especially among high risk patients. In the field of cardiothoracic surgical practice, several risk assessment tools or models, including the EuroSCORE II, have been proposed and developed by researchers based on clinical databases selected from specific populations ^8^. Concurrently, the EuroSORE II has become one of the most commonly used risk evaluation tool in many cardiac centres worldwide. However, it is crucial to note that the EuroSCORE II was actually developed based on data from mainly European countries ^9^. Therefore, the application of EuroSCORE II in other populations might need cautious clinical consideration as there are other inter-related factors such as genetic background of the population, different healthcare systems as well as different social and cultural practice.

In our present study, we have determined both the calibration and discriminatory power of the EuroSCORE II in our local population undergoing CABG surgery. Calibration of a model includes the determination of its ability to compare the predicted outcome (EuroSCORE II) with the actual outcome (actual in-hospital mortality) in the entire sample. Discriminatory power is the ability of the EuroSCORE II to distinguish patients who were still alive or who died in the hospital. Results showed that the EuroSCORE II has reasonable and fair calibration and discriminatory power in this group of Malaysian patients who underwent CABG surgery.

Many studies have been conducted around the world to examine the validity of the EuroSCORE II in predicting in-hospital mortality post-CABG. First of all, one of the most important findings in our study will be the in-hospital mortality rate. According to multiple validated studies that had been conducted across Europe, it showed a mortality rate ranging from 4.85%, 3% and 3.7% in Italy ^10^, Greece ^11^ and Serbia ^12^, respectively. Consistent with previous literature, we observed an actual in-hospital mortality rate of 4.7% in this study.

A multicentre prospective validation study was done to compare the EuroSCORE and EuroSCORE II among 4000 patients undergoing cardiac surgery in Spain. Results showed that both the EuroSCORE and EuroSCORE II has good discriminatory power (AUC > 0.75). In addition, the original EuroSCORE tends to over-predict mortality while EuroSCORE II under-predict mortality ^13^. Similarly, a single centre validation study in Hungary has also shown that EuroSCORE overestimated the risk of mortality while EuroSCORE II underestimated the risk. Despite that, EuroSCORE II was still better than its original version in terms of discriminatory power ^14^. Our study results showed a skewed distribution of EuroSCORE II and the median was 2.06 in comparison to the actual mortality rate of 4.7%, which certainly showed underestimation of risk by the EuroSCORE II.

In terms of calibration and discriminatory power, most of the validation studies in Europe including Spain, Italy, Greece, Serbia and Hungary has an AUC of more than 0.7, which indicates good discriminatory power and calibration ^10– 14^. However, there was a collaborative study between two centres in the Netherlands and United Kingdom, which showed that EuroSCORE II was not good in predicting mortality in patients undergoing cardiac operation. It showed an unsatisfactory AUC of 0.67, indicating poor discriminatory power. Particularly in middle-eastern countries, a slightly different results were observed. For instance, in Pakistan, it was shown that, despite having a satisfactory discriminatory power, EuroSCORE II was poorly calibrated and the original EuroSCORE actually fared better than the EuroSCORE II among isolated CABG patients in their local population ^15^. This can be attributed to various demographic-related factors or even study bias. Among our population of CABG patients, we observed an AUC of 0.7, which is deemed to be satisfactory in predicting in-hospital mortality.

Our study had shown that only female gender, age more than or equal to 65 years old, serum creatinine more than 120 micromole/litre and longer ICU stay are significant predictors of in-hospital mortality in patients post CABG surgery. In this context, independent variables were selected in line with the principle of parsimony so that our analysis can be more consistent and limited to as few variables as possible in the prediction model.

According to previous literatures, increasing age was found to be a significant risk factor by a few studies to investigate age as a risk predictor in patients undergoing CABG surgery ^16, 17^. In terms of gender, multiple studies had shown that female gender was an independent predictor for early and late mortality after cardiac operation ^18– 20^. Chronic renal dysfunction has also been known to have close association with mortality after CABG. After the establishment of EuroSCORE in 2003, a study was performed to look into patients undergoing CABG with a preoperative serum creatinine <200 µmol/L. It was shown that both the in-hospital mortality rate and stroke rate for this group of patients went up to 2.5%. Furthermore, the mortality rate also increased with increasing preoperative serum creatinine level ^21^.

Risk prediction is a very important area in cardiothoracic surgery that can serve to further refine the quality of patient care. By taking into consideration a series of relevant risk factors, the predicted risk by EuroSCORE II can guide us as to whether to perform an operation or to treat conservatively certain patients. Given the fact that multiple studies had shown that the original EuroSCORE was outdated and not applicable for risk prediction ^7, 22^, EuroSCORE II can replace its predecessor as a risk prediction model for mortality prediction. As discussed previously, a significant number of cardiac centres around the world including Europe, Asia and the Middle-East had validated EuroSCORE II with acceptable results. We believe that it can serve as a practical tool for the benefits of cardiac surgeons in terms of risk analysis, quality assurance as well as cost consideration.

Nonetheless, we do not deny the fact that it is still virtually impossible to develop an ideal risk evaluation model that fits everyone in the world as all of the models were developed based on clinical data from certain region-specific population. Moreover, given that cardiac surgery has gone through major advancement over the years in terms of improvement in surgical techniques and perioperative care, preoperative risk prediction has been shown to be a moving target that is both important and challenging to tackle.

Looking forward, our efforts for improvement will focus on the universality and practicability of the risk evaluation model. First of all, the lack of parsimony is a problem with the EuroSCORE II, which consists of 18 variables. A simpler risk prediction model with fewer variables that is able to predict in-hospital mortality would be better ^23, 24^. Should we be able to develop a relatively simpler and straightforward risk model in the future, the aim will be to have it provide the same predictive power but also be more user-friendly. Following that, we also recommend that a multicentre large scale study should be undertaken to incorporate population groups from all over the world with more variation in terms of genetic and social backgrounds so that a universal and culturally sensitive risk assessment model can be developed in the future. 

## Limitations

This study was limited by its nature of retrospective study. There was a considerable amount of missing data, which might lead to a relatively smaller sample size in performing logistic regression analysis on various independent risk factors. Due to its retrospectivity, patients with specific risk groups cannot be intentionally selected. In our case, we observed a skewed distribution of patients in terms of risk group (more than 90% of our patients are within the low risk group of 0-10%). In addition, Peterson *et al*. ^4^ from the Duke Clinical Research Institute had looked into the association between surgeon experience and mortality post-CABG. It was shown that surgeon experience was a significant predictor of mortality. The highest mortality rate was observed when patients were treated by low-volume surgeons. This study was conducted in a cardiac centre with surgeons with varying levels of surgical experience. That might directly or indirectly affect the outcome of surgery or even in-hospital mortality to a certain extent.

## Conclusion

This single centre large validation study showed that the EuroSCORE II exhibits reasonable and fair discriminatory power and calibration in predicting in-hospital mortality risk among patients undergoing CABG surgery in Malaysia. Despite being a single centre study and therefore may not be representative of the entire population, we think that it can be safely used as a risk assessment tool with cautious clinical consideration being applied on an individual basis.

## Data availability

Raw data for the study ‘Validation of EuroSCORE II in patients undergoing coronary artery bypass grafting (CABG) surgery in Malaysia’ are available both in excel and SAV formats. Data analysis is available in SPV format (SPSS output).

Dataset 1 – Demography and EUROScore II variables (Excel) 10.5256/f1000research.14760.d202215 ^25^

Dataset 2 – Demography and EUROScore II variables (SPSS) 10.5256/f1000research.14760.d202216 ^26^

Dataset 3 – SPSS Output 10.5256/f1000research.14760.d202217 ^27^

## Ethics approval

All research procedures were done in accordance with the ethical regulations set by the IJN ethics committee, Monash University Human Research Ethics Committee (MUHREC) and it abides with the Helsinki Declaration revised in 2013.
