# Supplementary material for: Validation of EuroSCORE II in patients undergoing coronary artery bypass grafting (CABG) surgery at the National Heart Institute, Kuala Lumpur: a retrospective review
Source: F1000Res. 2019 Sep 10;7:534. Originally published 2018 May 2. [Version 2] doi: 10.12688/f1000research.14760.2 (PMC7459866; doi:10.12688/f1000research.14760.2)
Supplement: Peer review history

# Peer review: Reviewer response for version 2

**DOI:** 10.5256/f1000research.22575.r69984

**Published:** 2020-08-28

**Authors:** Louise Y. Sun

**Affiliations:** 1 Division of Cardiac Anesthesiology, University of Ottawa Heart Institute, Ottawa, ON, Canada; 2 Institute for Clinical Evaluative Sciences, Toronto, ON, Canada

Musa and colleagues validated the EuroSCORE II in a retrospective cohort of 1718 patients who underwent cardiac surgery at a single centre in Malaysia. They concluded that the EuroSCORE II had acceptable discrimination and calibration at this large centre.

 Thank you for the opportunity to review this carefully constructed work. My comments mainly pertain to the clarity of reporting and the quality of data used:

1. Selection criteria: why exclude patients with repeat interventions during the same surgical admission? These patients would be at the highest risk for mortality, and including them may improve the predictiveness of the model.
2. The title and introduction suggest this study was about CABG patients. However, outcomes of “isolated CABG, isolated non-CABG, 2-procedures, 3-procedures, surgery on the thoracic aorta” were studied. In addition, why were patients who underwent isolated valve procedures not included?
3. Was the study data collected prospectively or retrospectively, and by whom? How accurate and reliable was the data? What was the degree of missingness, and how was this handled? Data quality could have an impact on the performance of the predictive model.
4. Statistical analysis: Do the authors mean multivariable (instead of “multiple”) logistic regression?
5. Looks like the weighted analysis is actually unweighted subgroup analysis.
6. Table 1 should also provide the characteristics of those who died vs. those who did not.

Is the work clearly and accurately presented and does it cite the current literature?

Yes

If applicable, is the statistical analysis and its interpretation appropriate?

Yes

Are all the source data underlying the results available to ensure full reproducibility?

Yes

Is the study design appropriate and is the work technically sound?

Yes

Are the conclusions drawn adequately supported by the results?

Yes

Are sufficient details of methods and analysis provided to allow replication by others?

Partly

Reviewer Expertise:

Big data, cardiac surgery, predictive modeling

I confirm that I have read this submission and believe that I have an appropriate level of expertise to confirm that it is of an acceptable scientific standard, however I have significant reservations, as outlined above.

# Peer review: Reviewer response for version 1

**DOI:** 10.5256/f1000research.16058.r48306

**Published:** 2019-05-13

**Authors:** Domingo Marcolino Braile

**Affiliations:** 1 University of Campinas, Campinas, Brazil; 2 Rio Preto State Medical School, São José do Rio Preto, Brazil; 3 Editor in Chief, Brazilian Journal of Cardiovascular Surgery, São José do Rio Preto, Brazil

Dear authors,

 I appreciated your manuscript very much.

 Congratulations for all the details that you presented in the work. I had some suggestions, regarding the sample that was analyzed in the work, considering that the majority of patients in the sample belong to classes I or II of the NYHA, and as you declare in the limitations, “more than 90% of our patients are within the low risk group of 0-10%”. Your work could have the advantage to be done in one single service, with the same quality of the surgery. Unfortunately, you said that the performance of the surgeons is not the same, between them.

 Particularly, I have critiques about the multicenter studies, which have a lot of qualities, but the surgeons from different services and nations have different results, like you expressed in the limitations. The problem of the work being retrospective brings some problems, but this does not compromise the results.

 Your discussion was very clear, adding your article in the present context of the literature. The conclusions are simple and straightforward, allowing the Cardiac Surgeons of Malaysia to use the EuroSCORE II, securely in your country.

Is the work clearly and accurately presented and does it cite the current literature?

Yes

If applicable, is the statistical analysis and its interpretation appropriate?

Yes

Are all the source data underlying the results available to ensure full reproducibility?

Yes

Is the study design appropriate and is the work technically sound?

Yes

Are the conclusions drawn adequately supported by the results?

Yes

Are sufficient details of methods and analysis provided to allow replication by others?

Yes

Reviewer Expertise:

Cardiovascular Surgery, Emeritus Professor at FAMERP and UNICAMP. Editor in Chief of Brazilian Journal OF Cardiovascular Surgery [www.bjcvs.org]

I confirm that I have read this submission and believe that I have an appropriate level of expertise to confirm that it is of an acceptable scientific standard.

# Peer review: Reviewer response for version 1

**DOI:** 10.5256/f1000research.16058.r33778

**Published:** 2018-06-13

**Authors:** Hasanat Sharif, Irfan Qadir

**Affiliations:** 1 Section of Cardiothoracic Surgery, Department of Surgery, Aga Khan University Hospital, Karachi, Pakistan; 2 Resident Orthopedics, Ghurki Trust Teaching Hospital, Lahore, Pakistan

The authors have used same methodology as we did in our study. This is the usual standard.

1. Please share details of two and three procedures whether they were CABG AVR or CABG MVR or DVR!
2. It would be a better idea to analyse mortality in performance of Euroscore II for isolated CABG and combined procedure separately.
3. Table 3 there is concordance between expected and observed mortalities in subgroups 0-10% and 11-20%. However for >20% there is gross mismatch of expected and observed mortalities. Please share details of 8 patients who died in this group. Did they belong to isolated CABG category or the combined procedure? As suggested above, please separate analysis of performance of Euroscore II for isolated CABG and combined procedures. This will give a true picture of performance in these two distinct groups. It may also explain the reason for underestimation in >20% group and may even improve concordance within results.

Is the work clearly and accurately presented and does it cite the current literature?

Yes

If applicable, is the statistical analysis and its interpretation appropriate?

Partly

Are all the source data underlying the results available to ensure full reproducibility?

Partly

Is the study design appropriate and is the work technically sound?

Yes

Are the conclusions drawn adequately supported by the results?

Partly

Are sufficient details of methods and analysis provided to allow replication by others?

Yes

Reviewer Expertise:

Clinical outcome research

We confirm that we have read this submission and believe that we have an appropriate level of expertise to confirm that it is of an acceptable scientific standard, however we have significant reservations, as outlined above.

# Response

**Published:** 2018-06-21

**Authors:** Ahmad Farouk Musa

**Affiliations:** Monash University Malaysia, Malaysia

Dear Prof Hasanat, thank you for your comments. My answers were detailed below accordingly.

1. Please share details of two and three procedures whether they were CABG AVR or CABG MVR or DVR

In our study, there are 160 patients who underwent a combination of two procedures, which included CABG + AVR, CABG + MVR as well as CABG + Aortic Root Replacement. For 33 patients who underwent three procedures, it included CABG+MVR+Aortic Root Replacement; CABG+MVR+AVR; as well as CABG + ASD Closure + Devega’s Tricuspid Annuloplasty.

  

1. It would be a better idea to analyze mortality in the performance of Euroscore II for isolated CABG and combined procedure separately.

Thank you again for your suggestion. We have already performed subgroup analysis based on weightage of procedures. Among 1488 patients who underwent isolated CABG, we observed an actual in-hospital mortality rate of 3.9% and a median EuroSCORE II (predicted mortality) of 1.918, which showed an underestimation of risk. Hosmer and Lemeshow Goodness-of-Fit test showed a significant p value of 0.032, indicating a significant difference between expected and actual mortality among this group of patients. Discriminatory power as shown by the ROC curve analysis showed an area of 67.8%.

 Among 160 patients who underwent two procedures, we observed an actual in-hospital mortality rate of 10% and a median EuroSCORE II (predicted mortality) of 3.712, which showed an underestimation of risk. However, Hosmer and Lemeshow Goodness-of-Fit test showed a non-significant p-value of 0.591, indicating no significant difference between expected and actual mortality among this group of patients. Discriminatory power as shown by the ROC curve analysis showed an area of 63.4%.

 Lastly, among 33 patients who underwent three procedures, we observed an actual in-hospital mortality rate of 18.2% and a median EuroSCORE II (predicted mortality) of 6.2, which showed an underestimation of risk. Similar to two procedures, Hosmer and Lemeshow Goodness-of-Fit test showed a non-significant p-value of 0.575, indicating no significant difference between expected and actual mortality among this group of patients. Discriminatory power as shown by the ROC curve analysis showed an area of 67.9%.

  

1. Table 3 there is concordance between expected and observed mortalities in subgroups 0-10% and 11-20%. However, for >20% there is a gross mismatch between expected and observed mortalities. Please share details of 8 patients who died in this group. Did they belong to isolated CABG category or the combined procedure?

Among the 8 patients who died in the 11-20% subgroup, 4 of them had isolated CABG (50%), while 2 of them underwent two procedures (25%) and the other 2, three procedures (25%) respectively.
